# Health mediation: an intervention mode for improving emergency department care and support for patients living in precarious conditions

**DOI:** 10.1186/s12913-023-09522-4

**Published:** 2023-05-16

**Authors:** Riwan Naït Salem, Michel Rotily, Themistoklis Apostolidis, Sophie Odena, Aurore Lamouroux, Célia Chischportich, Nicolas Persico, Pascal Auquier

**Affiliations:** 1 Laboratoire de Psychologie Sociale (LPS), Aix Marseille Université, Aix-en-Provence, France; 2 Centre de Santé Universitaire - Espace Santé Aygalades - Assistance Publique Hopitaux de Marseille, Marseille, France; 3 Centre d’Etudes et de Recherche sur les services de santé et la qualité de vie (CEReSS), Aix Marseille Université, Marseille, France; 4 Laboratoire d’Economie et de Sociologie du Travail (LEST-UMR 7317), Aix Marseille Université, Aix-en-Provence, France; 5 SCOP Regards Santé, Marseille, France; 6 Service d’Accueil des Urgences Adultes, Hopital Nord, Assistance Publique Hopitaux de Marseille, Marseille, France

**Keywords:** Health mediation, Emergency department, Frequent health service users, Precariousness, Vulnerable populations, Case management

## Abstract

**Background:**

Severe overcrowding of emergency departments (EDs) impacts the quality of healthcare. One factor of this overcrowding is precariousness, but it has rarely been considered a key factor in designing interventions to improve ED care. Health mediation (HM) aims to facilitate access to rights, prevention, and care for the most vulnerable persons and to raise awareness among healthcare providers about obstacles in accessing healthcare. We here present the results of an ancillary qualitative study to explore the prospects regarding a health mediation intervention implemented in EDs for deprived persons who are frequent ED users, from professionals’ and patients’ perspectives.

**Methods:**

Design, data collection, and data analysis were done according to a psychosocial approach, based on thematic content analysis and semi-structured interviews of 16 frequent ED users and deprived patients exposed to HM and of 14 professionals in 4 EDs of South-eastern France.

**Results:**

All patients reported multifactorial distress. Most of them expressed experiencing isolation and powerlessness, and lacking personal resources to cope with healthcare. They mentioned the use of ED as a way of quickly meeting a professional to respond to their suffering, and recognized the trustworthy alliance with health mediators (HMrs) as a means to put them back in a healthcare pathway. The presence of HMrs in EDs was appreciated by ED professionals because HMrs responded to requests they were not able to access and were perceived as an efficient support for caring for deprived persons in emergency contexts.

**Conclusions:**

Our results are in favour of health mediation in EDs as a promising solution, requested by patients and ED professionals, to cope with frequent ED users and deprived patients. Our results could also be used to adapt other strategies for the most vulnerable populations to reduce the frequency of ED readmissions. At the interface of the patients’ health experience and the medico-social sector, HM could complete the immediate responses to medical needs given in EDs and contribute in alleviating the social inequalities of health.

## Background

Precariousness is a complex and multifactorial concept, both in its dimensions and its causes, affecting heterogeneous populations [1]. It can be defined as “a state of social instability characterized by the absence of one or more safeguards that allow persons […] to enjoy their fundamental rights» [2]. Thus, this definition calls upon several components: economic, social, professional, cultural, psychological, and health [3]. It can be transitory without being in the same temporality as poverty [4]. Precariousness affects the entire population along a social gradient. The most precarious persons are directly concerned by increases in social inequalities in health defined as the systematic, avoidable, and unfair differences in health outcomes between populations and between social groups within the same population, or as a gradient across a population ranked by social position [5]. The causes of social inequalities in health are multiple, and their health effects are major [6, 7]. Thus, in France, although health outcomes are among the best in the European Union, social and geographical health inequities persist and even tend to increase [8, 9]. This raises the question of how to build a more equitable healthcare policy and manage health issues with the persons living in precarious conditions, especially in emergency situations and dedicated services.

### Precariousness, a major contributor to the repeated use of emergency departments

Iterative use of Emergency Departments (EDs) results in lost opportunities for patients and strongly impacts the finances and the workload of EDs, with severe overcrowding resulting in long waits that affect the quality and efficiency of care [10, 11]. Several risk factors for frequent ED readmissions have been identified, such as age (younger or older, according to studies), lacking a partner, chronical diseases and comorbidities, heavy users of general practice services, other primary care services, and other hospital services, having psychiatric problems or related to alcohol, economic hardship, being unemployed or dependent on government welfare, being under guardianship, being uninsured, living closer to ED [12–17]. Many of these risk factors could be targeted by social interventions and personalizing/coordinating care, and improved by a better education and empowerment of vulnerable patients. Although the results of several studies support the effectiveness of some strategies to reduce the readmission rate of frequent ED users, their global effectiveness is still under debate, mainly because of their heterogeneity (ED definition, the strategies tested) [18–20]. Several systematic reviews suggested that targeted interventions, particularly case management, could reduce ED visits [18, 19, 21, 22] and be cost-effective [23], but few targeted specifically vulnerable patients [20–22]. Interventions implemented were more often case management, sometimes care plans and rarely diversion strategies to non-urgent care, printout case notes or social work home visits. Case management involve multi-disciplinary teams, including physicians, nurses, psychologists, social workers and/or housing and community resource liaisons, who developed tailored care strategies for patients and linked them to necessary services [24]. Grazioli et al. proposed a protocol for implementation of case management in the Swiss health care system [24]. Such protocols will have to be adapted to the different healthcare systems, especially in the current context of overcrowded EDs that are also facing a shortage of doctors and nurses, in France as in other countries [25–27]. Little is known about the impact of CM on deprived frequent ED users and interventional trials in this population are crucial.

### Health mediation to reduce health inequities

In France, improving health policies for vulnerable and deprived persons is mainly based on improving social rights to allow these persons to have financial access to healthcare, but insufficiently in terms of case management, health literacy and inclusivity [28]. Many persons do not know their rights, and when they do, they do not know how to assert them; it is also established that giving up on care has causes other than mere finances [29]. At the end of the 1980s, a new form of care, called health mediation (HM), has appeared aiming at reducing social health inequities, especially for patients living with chronical diseases, including AIDS, and mental diseases [30]. First, HM is not intended as an intervention to prevent or solve medical disputes and conflicts [31]. HM is intended to be a proximity interface aiming, on the one hand at access to rights, to prevention and to care for populations presenting various factors of vulnerability that distance them from the health systems, and on the other hand at raising awareness of the actors of the health system to the specificities of these populations and to the obstacles they encounter in their healthcare pathways [30]. HM contributes to the opening and continuity of health coverage rights, access to care, and the reception of persons [32]. HM is based on the major principles of “going towards” populations, health and social professionals and institutions, and “doing with” in a logic of empowerment of individuals [30, 33]. The “going towards” approach has two components: (1) physical movement, “outside the walls”, towards the places frequented by underserved populations on the one hand and towards health professionals or institutions on the other; (2) openness towards others, towards the person as a whole, without judgement, with respect [33]. In some countries, work has been done with professionals close to health mediators (HMrs), such as community health workers (CHW) or community counsellors (CC), family welfare volunteers, community nurse auxiliaries, health surveillance assistants and matrons, highlighting the benefits of their interventions in hospitals [34, 35]. A systematic review showed that programs involving CHW promote more equitable access and can contribute to improved uptake of referral for health facility services, and underlined the needs for equity during planning and implementation of such programs [35]. A meta-analysis suggested that integrating CHWs into chronic care management may reduce care utilization and costs [36]. In some ways, HMrs are close to CHW, in the sense that they serve as a link between health/social services and the community to facilitate access to services and improve the quality of service delivery; they also build individual and community capacity by increasing health knowledge and self-sufficiency through a range of activities such as outreach, education, informal counselling and social support. On their part, HMrs are not always trusted members of the community served or the neighbourhoods, and they do not provide education, support nor advocacy at a community level but only individual. Unlike the community counsellors (CC), HMrs do not offer mental health services nor therapy for individuals. Like CC, however, they give support to individuals in difficulties and low health literacy. In France, several settings have implemented HM to improve the management of chronic and mental illnesses [30, 37, 38]. Long lacking a legal framework and professional benchmark, HM has been officially recognized in France by the law of modernization of the healthcare system in 2016, and defined in 2017 by the French National Authority for Health [33], as a set of actions to improve access to rights, prevention, and care, in order to promote health and thus move towards greater equity. Although HM has been widely promoted by the French Ministry of Health and many actors in the healthcare system, and its implementation has been evaluated in the context of health promotion and access to health in vulnerable people, tangible data on its effectiveness on access to health, quality and efficiency of health care in these populations are not available [30, 38–40]. Although it seems entirely appropriate in the context of deprived frequent ED users, HM has never yet been tested or even evaluated in this context.

### A need for tools adapted to deprived frequent users of emergency departments

Very few studies have reported on the impact of CHW on frequent ED users. Though, CHW could help leverage EDs as an entry point into the healthcare system [41]. CHWs implemented in EDs can offer healthcare screenings and education, care coordination for a vast number of health conditions [41, 42]. Among the ED “super-users” working with CHWs, the care coordination intervention demonstrated a decrease in costs per patient for the EDs [43]. Having a CHW service embedded in the EDs workflow could allow for patient-centric care to improve overall health outcomes and elevate some of the emergency physicians’ responsibility in ensuring proper follow-up to a variety of medical services [41]. Emergency physicians and most of nurses do not have specific training nor the time to determine pyschosocial needs and most do not know what resources are out in the community to fill in the gaps. At present, neither EDs nor their staff are equipped to deal with the complex needs of frequent users of EDs [44]. After the examinations, care and recommendations have been made, deprived patients are discharged from EDs with a report to their general practitioner (which they do not always have), one or more prescriptions for tests or medication that they do not always understand or that they do not know how to carry out, cannot or do not want to carry out, do not identify as priorities. Social services are attached to EDs but are not sufficiently staffed and trained in empowerment, care pathways, health literacy and outreach techniques. HM could respond to patients’ needs as they express it, respect their need for control over the situation, promote their ability to make their decisions, strengthen their sense of self-efficiency and their motivation to healthcare use. HM could also strengthen the ability to make decisions favourable to health in a logic of empowerment, and reinforce people’s perception of the healthcare benefits [40]. A HM intervention targeting deprived frequent ED users, starting in ED and consisting of education actions and navigation in care system could reduce readmissions to ED.

To develop additional measures to alleviate social inequalities in health, we have set up a project (entitled M2PRUSSE, see below) whose main objective is to evaluate the effectiveness of HM in EDs on the 90-day readmission rate, in deprived and vulnerable patients. Here we present the results of an ancillary qualitative study of the M2PRUSSE project aiming to explore the prospects regarding a health mediation intervention implemented in EDs for deprived persons who are frequent ED users, from professionals’ and patients’ perspectives.

## Methods

### Context of the M2PRUSSE study

The study enrolled patients who met the inclusion criteria (admitted in one of the four participating EDs, one visit to the same ED in the 90 days prior to inclusion, or twice in the last 6 months, or three or more times in the last 12 months (Study NCT03660215); to have an EPICES social precariousness score higher than 30. The EPICES score estimates the level of precariousness by the means of 11 binary items: two are related to material deprivation, six to social deprivation, one to health and financial difficulties, two to precariousness scales. It can vary from 0 (no precariousness), to 100 (extreme precariousness), 30 being the cut-off point to categorize people as in a precarious situation [6]. Two EDs were in densely populated urban areas with high levels of precariousness (Hôpital Nord and Hôpital Européen, in northern and central districts of Marseille); the two other EDs were in less urbanized areas characterised by pockets of neo-rural precariousness (Arles and Manosque). The 726 patients included were randomized at the time of their visit to the ED into one of two arms: “control” (usual care) or “experimental”. In the experimental arm, the patient was managed by one HMr from the time of admission to the ED and followed up for 90 days, according to the recommendations of the French High Authority for Health [33]; examples of HM are displayed in Table 1. All patients were documented for the reason and severity score of the ED visit, the main and associated pathologies, the quality of life (WHOQOL-Bref), and the mode and reason of discharge. The inclusion of patients in the M2PRUSSE project started in February 2019, and the last patient was included in November 2021. The last follow up ended in May 2022.

**Table 1:** Examples of health mediation (HMr: Health Mediator; ED: Emergency Department)

Case 1: Mrs G, 74 years old, came to the ED several times in the last six months for the same reason, a high blood pressure. She reported regular meetings with the GP and the cardiologist, with a nurse visiting every day to check her blood pressure and medications. Mrs G and her husband were both on full social security.	
The couple live on a small pension. At the time of the initial interview by the HM, Mrs G stated that she had been having financial problems for 3 months due to the suspension of housing allowances. The husband had been to the family benefits fund several times, but had not been able to resolve the problem. The loss of support meant that the couple’s resources were significantly reduced, making it difficult for them to pay the rent or buy food. This situation was a great source of stress for Mrs G.	
The HM put the couple in touch with a social worker to look for a solution regarding housing benefits and to find social housing. After several weeks, the couple recovered their housing benefit. In the meantime, the HM informed Mrs G. about the various food distribution associations near her home and called her regularly to check on her and reassure her. Mrs G. did not return to the ED in the following months.	
CASE 2: Mr T., aged 58, regularly returns to the ED for several reasons (chronic bronchitis, depressive syndrome, alcohol abuse). He is very isolated, out of touch with the health care system, he has neither a regular general practitioner, nor complementary medical insurance, nor exemption from fees for a long-term illness. Mr T. does not receive housing benefit because he has no rental agreement and pays a low rent in an unhealthy dwelling in danger of collapsing. Mr T. has a very low income, receives financial allowance for his disability. He has a large debt with the hospital and refuses any contact with a social worker.	
The mediation lasted 90 days. Several meetings took place at home and at the hospital, with dozens of telephone calls. The patient was reintegrated into a care programme with a GP in his neighbourhood, obtained recognition of his chronic pathologies for full healthcare insurance, and the hospital’s litigation department was informed in order to regularise his debts. He obtained help with the payment of supplementary health insurance and was able to start dental and ophthalmic treatment. Finally, he was evacuated from his home and rehoused, and a social follow-up with an association was launched. A neighbourhood citizens’ association keeps in touch with Mr T. to break his isolation. The HM had to call on several structures outside the hospital to find the best alternatives for his complex situation. Mr T. reduced the number of ED admissions from 15 in the 6 months prior to inclusion to 4 in the 90 days following inclusion.	

The five HMrs were full-time, paid employees with a 2- to 5-year degree after the baccalaureate, and a diploma in social work, with basic experience in the healthcare sector; medical backgrounds were not required. Qualified applicants were selected based on good communication skills, good knowledge of social rights and procedures, and of common health care pathways, on abilities/experience to teamwork and networking with health/social professionals inside/outside EDs, and to manage relationships with deprived persons with respect to ethics and equity.

They were initially trained by a professional in HM for vulnerable and deprived persons (including attitudes and behaviour with these persons). They were supervised by an HMr with 10 years of experience and a general practitioner, with routine group or individual meetings to present challenging cases.

The tasks of HMrs consisted of (1) administering a questionnaire on socio-demographics, quality of life, health literacy, and reasons of admission to ER; (2) evaluating the socio-medical needs of patients according to ad hoc guidelines; (3) defining objectives corresponding to activities and resources of the services requested; (4) accompanying persons towards prevention and care, and helping them to understand the access to social and health care; (5) acting as an interpreter and bridge to the persons concerned but also to health professionals and social workers; (6) adopting a benevolent stance and active listening in order to detect individual and collective problems that might require specific information or prevention. All these tasks are carried out with a view to improving the capacities for health empowerment of patients under care.

### Data collection

We designed, collected and analysed data according to a psychosocial approach based on a thematic content analysis [45–47] of semi-structured interviews in order to give the interviewees the opportunity to develop and direct their comments, while integrating different themes as they spoke. These interviews were conducted by one social health psychologist, on the basis of a guide developed through five pre-exploratory interviews. Within this framework, we wanted to take into consideration the experiences and perceptions of the support situations, the expectations and results obtained, and finally the levers and barriers in the implementation of HM. The guides began with an introductory phase, initiating the interview with general questions about the experience of ED care. It ended with a closing phase during which the interviewee was offered a summary to elaborate on elements that had not been addressed during the interview. Two guides were adapted, one for professionals and the other for patients.

Before the interviews, each participant was told that the interview would be anonymous and confidential, and that the content would be shared only with persons involved in the study. Participants were explained that the interview would be recorded for transcription purposes. The duration of these interviews was estimated to be thirty minutes. The professionals were asked to meet at their place of work, and the patients chose the site (home, outside or -for Marseille only- a health centre). Verbal informed consent was obtained before starting. Sociodemographic information was also collected at the end of each interview.

#### Patients

We contacted the patients by telephone. This first contact allowed us to present the study to them, to collect their consent and, finally, to propose an appointment for the research interview. In the first phase of the interviews, the patients talked about their experience of the HM and the support offered to them. Following this phase, their perceived effects of this support were discussed, as were their expectations and needs. A final phase focused on the representation of HM and possible improvements.

#### Professionals

The objective was to collect points of view from different professional positions available in EDs, as regards health care and the HM intervention. In the first phase, the professionals talked about their experience of the support offered to patients, as well as their perception of the HM intervention. Based on the perceived effects of their interventions, they could propose adjustments to meet the patients’ demands. Finally, they related their experience of the implementation of the intervention, of the perceived effectiveness and feasibility.

All the interviews were conducted in April 2021 by a single social psychologist who immersed himself in the partner ER to meet the teams and understand their organisation. This served to present the study process and to identify future participants, on a voluntary basis.

### Study population

Professionals and patients were recruited from the EDs of four South-eastern French hospitals participating in the M2PRUSSE study (see above). Fourteen interviews with professionals and sixteen interviews with patients were conducted:

#### Patients

The objective was to recruit 15 patients. From the list of the patients enrolled in the M2PRUSSE study who had ended their follow-up with the HMrs during the last 9 months, we randomly selected a first batch of 35 patients. As several phone numbers were unassigned or patients remained unreachable or refused to participate, a second then a third batch of 35 patients were randomly selected until to reach the expected number of patients accepting the interview. At the end of this process, we attempted to reach the 105 patients; 31 telephone numbers were unassigned (30%), 21 patients remained unreachable (20%) despite at least three messages left on their answering machines; 53 patients answered the call or were called back later (50%). A total of 16 patients agreed to participate in the interviews, including 7 women and 9 men.

#### Professionals

Fourteen professionals were interviewed: 11 women and 3 men, including 3 HMrs who had followed patients included in the M2PRUSSE project, 3 agents in charge of receiving patients and performing administrative registration, 3 nurses, 1 physician, 2 mediation officers (linking the emergency care team, the patients, and their relatives in the waiting room), 1 social worker, 1 hospital service agent (in charge of room cleaning and preparation, dish delivery and stretcher bearing).

### Data analysis

All interviews were recorded and fully transcribed. One researcher performed a thematic content analysis, which explores the semantic discourse of all interviewees and implements systematic and objective procedures for describing the content of the interviews, aiming at inferring knowledge on production conditions [48]. For each interview, a first reading of the transcript identified the themes evoked through a floating reading [49]. The content was then broken down into units of meaning, themselves classified into different categories according to analogical groupings The aim was to identify the units corresponding to a theme in the discourse, which could be a word or a paragraph. Within each theme, the units were discriminated into sub-themes to specify the characteristics of the theme evoked by the persons, reflecting their social experience. This initial analysis - in themes and sub-themes - was subject to modification during its development. Themes that were not sufficiently addressed were removed to make the analysis clearer and to prioritize other themes that were more meaningful. The results were not shared or discussed with patients or ED professionals. We applied the methodological triangulation by linking the causal explanations of behaviours and the psychosocial interpretation of the meaning of the behaviours [41, 42]. The data collected and analysed by one researcher was shared and discussed with the scientific board composed of researchers and practitioners of different specialties (sociologists, health psychologists, emergency physicians and public health physicians); among them, only two have experienced HM in their practice or research. The researcher who collected, coded and analysed the data was involved neither in the main hypothesis of the study, nor in the choice of HM to limit the readmission rate among frequent ED users. We complied with the eight items of the domain 1 COREQ checklist, corresponding to the research team and reflexivity [50].

## Results

### Patients

The content analysis of the interviews with patients revealed four themes (Table 2).

**Table 2:** Summary Table of Thematic Content Analysis for Patients

Themes	Interview excerpts
The testimony of biopsychosocial distress	“And, even, health, plus administrative, I can’t. I can’t get anywhere. But I explained, but nobody listens to you, yeah nobody listens to you. Sometimes even, even the words they, I can’t find the words, the people they, they don’t try with you, and they let you down. Come on. He doesn’t speak well, come on. Forget it, come on, he knows nothing. But I have, I have there, despite sometimes, I can’t speak, even the words I can’t, I can’t find them” “The therapies I stopped because, either the doctor died, or he is closed. Because I went there, it’s in Marseille, and afterwards, X told me you have to look for another one, but it’s very far, it’s in (name of place). Then I, I, I didn’t go”
Health mediators: The importance of interpersonal and communication skills	*“Well, by talking, by listening to me, that’s it, by listening to people, she really understands the patient. She understands the patient, she understands what the patient is, she comes to help a patient, she doesn’t come to do business, I mean, not, I’m here, I work, that’s all, she was really there, she does it from the heart. It’s really a work of the heart, and she called me at home, when I went out and everything, she gave me advice, she directed me towards the thing there, frankly, yeah nothing to say frankly top “* *“X yeah she came, she’s, a saviour she […] she came to talk to me at first, said yes, pfff, there, how can I say it’s like everyone else, what, they come, afterwards I saw that frankly she was really, she’s nice, sweet, she cheered me up, fortunately she was there, I wasn’t well, fortunately she… I was not well, it’s a good thing she was there, she reassured me, she reassured me a little bit about the way things worked.”*
Feedback on the medico-social mediation project	*“It was a scheme that could help a lot of people, so it’s a good initiative. I would say 10 out of 10»* *“Because first of all, she knows how to take, how to deal with a patient, she encourages him, she is the one who encouraged us to call the CAF (*family allowance fund), *and everything, so Madame X is special, Madame X really is, very special, I tell you the truth “*
Health mediation: an opening to new resources	*“With my GP we wrote a letter, and I went to the hospital to make an appointment. They gave me an appointment, and then I continued with him […] I was with X, and as soon as X referred me to my GP I continued with him"* *“the girl is good, she’s nice, we spoke two or three times at the hospital, she comes and asks me questions, she shows me how to use the medication, there are ways, before I did it, but she explained to me how to use the Ventolin, how to put it, like this one, it’s good, sometimes we don’t know, before I took the Ventolin like this but it doesn’t go through, but she explained to me that I have to put it in an inclined position, after that, it’s very good”*

*The testimony of a bio-psychosocial distress: “I explained, but nobody listens to you, yeah nobody listens to you. Sometimes even, even the words they, I can’t find the words, the people they, they don’t try with you, and they let you down “, Patient 5.*

All patients reported multifactorial distress (Table 2). Their difficulties seemed to be reciprocally increased by individual, interpersonal, and situational characteristics. Most of the patients expressed a lack of resources and perceived the absence of social support as reinforcing their precariousness *(“We are lost. When I was, I was not followed with her, I don’t even know, you have to have a doctor already. It’s the first thing, but I don’t even know how to have a doctor […], I don’t know what these things are. She did all this with me”, Patient 2).* Social support appeared as a protective factor that seemed to promote access to resources and maintain a link between the patients and their environment. Some patients expressed an experience of isolation and powerlessness, so that the health difficulties became chronic. The latter seemed to be explained by non-use of care or non-adherence to treatment. In the same way, the lack of knowledge of the health care system was reported as leading to medical wandering. Many mentioned a lack of understanding of the health and social systems, due to difficulties in communicating and in assimilating the information *(“The therapies, I stopped them because, either the doctor died, or it’s closed. Because I went there, it’s close, and then, then, one told me you have to look for another, I found but it’s very far away. After I, I didn’t go”, Patient 6)*. However, this wandering could lead to new difficulties such as delay of care, evolution of the disease, and amplification of suffering. To compensate for medical wandering, several patients mentioned EDs were a way of quickly meeting someone to respond to their suffering *(“I’ve been here for 15 years, I’ve never found a help. Except like the hospital, that, that, yes.”, Patient 5)*.

*Health mediators: the importance of interpersonal and communication skills*: “*Well, speaking, listening to me, that’s it,.., she really understands what the patient is, she comes to help a patient, she doesn’t come to do business”, Patient 7.*

The patients have chosen EDs to obtain a rapid solution to their problems and seized this opportunity to meet with the HMrs. They recognized HMrs provided accompaniment on a case-by-case basis. The relational alliance was built through a relationship of trust. The time given, the listening, the empathy, and the attention were frequently mentioned as relational vectors *(“She took care of me, she advised me, it made me feel good, she called me from time to time when I was at home, she went to see the doctors”, Patient 11)* HM broke social isolation by proposing social support, which was sometimes limited until then. The relational alliance was perceived as a protective factor in the patient’s care pathway, since it provided resources that they could not access alone. In this context, the relational alliance and the follow-up were jointly reinforced throughout the proposed support *(“I contacted her several times, I said things, I’m fine […] she relieved me”, Patient 1).*

*Health mediation: an opening towards new resources*: “*she comes and asks me questions, she shows me how to use the medication, there are ways, before I did it, but she explained to me how to use the Salbutamol, how to put it”, Patient 12.*

The patients expected mediation to provide them with expertise and social support, in order to take the necessary steps to open their social insurance rights or to find answers to specific needs (e.g. finding an apartment) *(“a lady, she called me, she did the help for the insurance, yes, I did it there, she did it to me. After they stopped it, that’s not, that’s not normal, I did an interview with the lady, there it is, then they gave me the insurance”, Patient 15)*. Other patients were waiting for effective help to meet their primary needs (e.g. food, accommodation). The HMrs provided an interface between patients and professionals, acting as a reassuring relay for the people *(“I was with her, and as soon as she gave me the orientation with my attending physician I continued with him”, Patient 2).* The patients seized new this resource to develop personal and social skills. The patients described the way in which HM succeeded in favouring their empowerment and their proaction within their health and social pathway (e.g. making appointments, obtaining information, therapeutic compliance, etc.). They expressed difficulties with regard to the closure of the follow-up *(“When she reminds me, she tells me I didn’t get them, but you have to call this number. I call, but there’s nobody now, I told you, the door is closed for me», Patient 11)*. For several patients, the interruption of the research marked the end of the social support and resources provided until then. HM was perceived as a means of compensating for the inequity in health and the chronicity of the difficulties encountered.

*The feedback on the health mediation project: “It was a scheme that could help a lot of people, so it’s a good initiative. I would say 10 out of 10”, Patient 9.*

The patients mentioned the changes and the lack of changes they perceived since their participation in the program. Their satisfaction corresponded not only to the achievement of the objectives but also to the quality of the social link built between the HMrs and them (“*It’s true that there’s a lot of empathy for people, patience for that, she knows how to approach people, which is why I told you last week that I would praise you because I have a good memory of it.”, Patient 14).* The breaking of social isolation allowed by HMrs was recognized as having favoured their registration within the health and social systems. By allowing a dynamic favourable to change, HM was perceived as a means to recover a social place. The reinforcement, or even in some cases the creation of this link, allowed the expression of the expectations, needs, and frustrations of each person with regard to the system, but also, more broadly, concerning their life course *(“People they go by like that, interns they go by like that, not even they calculate us, she came in, she took care, she talked, she listened.”, Patient 7).*

### Professionals

The content analysis of the interviews with patients revealed four themes (Table 3).

**Table 3:** Summary Table of Thematic Content Analysis for professionals in EDs

Themes	Interview excerpts
The integration of health mediation within EDs	“The M2Prusse project aims to find out if people in precarious situations come back to the ED on a regular basis or not, and in particular, can we identify the patient’s problem and solve it, and finally, can a health mediator be put in place in the ED to solve this problem and, in particular, to relieve the congestion in certain situations and to help the health professionals in the ED?” “I saw that all these people, you took them, they need help because they have emergencies only, I think you were expected, you are, I don’t know of a good listening, their needs, me I think it was very good”
Roles of the health mediators (HMrs)	“I (HMr) can direct them to associations that help the patient to fill out files, everything that has to do with administrative procedures on the one hand, and institutions. I can also direct them, for example, to people who want to go to the Home for Disabled Persons, to structures that could help with health, or with the administrative aspect, so it can be a referral to the health authorities of the sector where the patient lives. I’m in contact with health care professionals, with people from the social security system, with the health care access offices (for patients with no social security), with health care units, etc.“
Responding to precariousness	“It’s not just about focusing on the medical symptoms but also on the social side. There are many patients who are decompensated because their personal and social situation has a huge influence on their state of health, and well, most of the management that is currently done focuses on the medication and the symptoms and not on the social aspect. Even the doctors don’t understand if the person is taking the right medication, why their blood pressure is decompensated. It’s really a medical treatment, it must be accompanied by a social treatment for it to be successful”
Implementation of health mediation	“It allowed us to highlight these people who use the ED, and to try to understand, with indicators that were worked on a little, something a little professional, to understand why they use it, what they come to find there, what their pathways are, what place the ED occupies in their pathway […]. …) they allowed us to hand over on situations that had been completely invisible until then, and through the passage to the ED and what was proposed as accompaniment in the framework of the m2 PRUSSE program, all of a sudden people became real, there was an outline, and there was, it allowed us to unravel incredible situations, and I think that it is like a magnifying glass what, it really allowed, that’s what I understand about the project”

The integration of health mediation in emergency departments “*I saw that all these people, you took them, they needed help because they had only emergency, I think you were expected”, Staff 3.*

Most of the ED professionals were involved in one or more stages of the implementation of the M2PRUSSE project and all were aware of the HM interventions. However, some of them were not aware of the project’s missions and did not participate much, either because of their workload or their lack of motivation. The support of the HMrs was valued because it satisfied requests/needs that they were not able to fulfil (e.g., opening of rights, referral to other professionals/facilities) *(“There is all that is social. We don’t necessarily have the answer, people come here, we don’t know what’s going on behind it in fact”, Staff 12)*. All agreed on the need to make the HMrs permanent in order to reinforce the social and relational aspects of care, by integrating them in the emergency care process *(“After that, it would be nice to have a person who would be there all the time, to focus on those people who come back all the time and for maybe times explain to them», Staff 5)*. Two timeframes seemed to fit this context: the HMrs had to put in place an accompaniment whose results would be perceived in the medium and long term while the carers were obliged to provide an effective response in the short term *(“They do a job, that’s too good, because they take, they take the time to see people, to take care of people, that’s it.”, Staff 3)*. Their missions with the patients, as ED professionals, were conducted in parallel with the activity of the services, in order to respond to different but interdependent problems. From this position arose a professional isolation, sometimes considered as strong by the professionals, especially in difficult situations that could have a strong psycho-emotional impact *(“I’m not trained to see people’s suffering like that actually…I end up in an emergency department seeing people who hurt every day, that’s not the prettiest thing to do”, Staff 1)*. The HMrs also explained that they did not have enough support to deal with these challenging and at-risk situations for their psychological health.

*The role of health mediators: “a role of intermediary, in capacity to be in connection with the professionals of all the fields, which is already a big challenge, to have the global vision, not to be in the silos of care”, Staff 10.*

The HMrs used their activity as a means of defining HM. Specifying their role during the interviews allowed them to elaborate and specify their identity, at the crossroads of several professional postures. From the point of view of the professionals interviewed, the different roles of the HMrs were based on the regulation of patients’ visits to the ED. Thus, one of their main objectives was to inform and (re)orient patients in order to reduce the use of ED *(It’s identifying people, how to say, precarious, to help them, to orient them, so they don’t always come back to the same place, you have to orient them in a specialized field “, Staff 9).* In addition, the HMrs developed an accompaniment through which they sought to reinforce the autonomy of patients. In this respect, they described the importance of the patients’ place in order to make them actors of their health and their care, and to reinforce their empowerment. To achieve this, the HMrs also aimed at health education, enhancing the level of health literacy and self-care skills (*I would define the role of health mediator as someone who accompanies the patient, because they are still patients for us, and towards access or a right to health whatever it may be, but it is really giving them the keys to take care of themselves as a priority.“ Staff 2).*

*Responding to precariousness: “There are many patients who are decompensated because their personal and social situation has a huge influence on their health”, Staff 1.*

The ED professionals perceived precariousness as a multifactorial phenomenon resulting from individual, relational, and/or situational problems. Some of them also considered that precariousness could be transitory or silent *(“They say they are not precarious because they have no financial needs, but finally when you talk with them, they are in a rather important social isolation”, Staff 1)*. The professionals explained that they identified the patients’ vulnerabilities during their treatment in the ED, before referring them to the HMrs. The weaknesses identified could concern health status, problems related to housing, financial resources, access to social rights, social isolation, and wandering in the healthcare system. This required HMrs to provide holistic support to address these interactional aspects of precariousness *(“The identification of difficulties that had led them to emergencies, the recognition of much broader difficulties, the identification of fragilities … to open the doors to other problems encountered”, Staff 14)*. The professionals indicated that many patients used the emergency services as a means of overcoming the difficulties they encountered in receiving care, of reducing waiting time for care, and of concentrating examinations in one place. Others, who did not have an attending physician, went to the ED to obtain regular follow-up and the assurance of being received by care providers. Still others preferred these services because they believed that care was free. The ED professionals reported that, overall, the patients were satisfied with HM *(“Patients often verbalize it, they say “it’s great to be accompanied”, if only to call, to check in», Staff 13)*.

*Implementation of the health mediation:”They allowed us to act on situations that had been completely invisible until then”, Staff 3.*

The HMrs emphasized that establishing a relational alliance from the beginning of the accompaniment was crucial for success. ED professionals presented the setting as another important feature for successful HM. For most professionals, this required the presence of the HMrs in the ED, and not in another place *(“A mediation would have to be permanent, it would be very expensive anyway, but in any case you also have to come at night, the day you have to come a lot on weekends too”, Staff 12)*. Another key factor for HM to succeed was the reinforcement of the network between HMrs, health professionals, and health and social structures in the surrounding area. The professionals described the importance of a multidisciplinary approach in order to respond effectively to patients’ needs. HMrs explained that this work could not be accomplished without a reciprocal and prior identification of the missions of each healthcare worker *(“I have an address book, I have a network because mediation cannot work alone”, Staff 13).* They thus expressed the need to know the professionals and structures, so as to use the resources they offered. The COVID 19 pandemic was reported as having an impact on the provision of support and the reinforcement of social isolation of some patients *(“The aggressiveness is going up, when we’re working with COVID. We’re not bringing families inside the ED, so there’s a lot of aggressiveness”, Staff 3; “people are getting lonesome, they have no listening, families are scattered”, Staff 4).*

## Discussion

This social-health psychological analysis identified the interest of HM for deprived persons who frequently use EDs. It showed that both these persons and professionals recognized the needs to take care of the bio-psycho-social distress and the utility of HM. Both patients and professionals were satisfied and wished to see HM perpetuated in the EDs. Beyond the expected initial objective of diminishing the number of readmissions of deprived persons, HM appears, from the perspective of both patients and professionals, as an opening towards new resources, as able to recreate a social link with the healthcare system and to contribute to a better healthcare pathway. From the point of view of ED professionals, HM supported their missions of healthcare and contributed to alleviating psychological suffering, and deserved to be extended to night hours, week-ends and lockdown periods, such as the COVID-19 pandemic. HMrs perceived the benefits of their interventions on patients and professionals to respond to precariousness and equity in health distribution, but they also highlighted psycho-emotional burden and isolation as difficulties in their professional well-being. Several key success factors of HM have been identified, such as a relational alliance based upon trust, interpersonal and communication skills, a perfect knowledge of surrounding resources and networking, an inclusive and empowerment approach. HM for deprived persons appears as a valuable intervention that could be adapted to other EDs.

Some methodological limitations are worth noting.The sample size is only 30 and we are not assured of the saturation of the collected data. Among the patients we tried to reach, 50% were unreachable, and among those reachable [51], 16/53 patients (30%) accepted to be interviewed. Unreachability is one of the main issues in working among deprived and homeless persons. As far as we know, few studies have focused on that issue. From our field experience, many deprived persons discontinue their telephone subscription because of financial issues, have technical difficulties to listen to their voice messages, and face constraints/barriers to respect their appointments. The COVID 19 pandemic also impacted the organisation and the burden of EDs and HM, and has enhanced the isolation of patients and the difficulties of remaining in contact with them. The HMrs were obliged to favour phone contacts instead of face-to-face meetings, and some patients were lost to follow up. This limitation due to the pandemic may have falsely overshadowed the patients’ situation and reinforced the usefulness of HM due to their increased isolation. This could also lead us to conclude the usefulness of HM in times of lockdown. Because of the work load of ED professionals in this pandemic and the loss of healthcare professionals because of illness, burn-out, and the refusal of mandatory vaccination, it was also difficult to convince professionals to spend time in interviews. However, the content of interviews was rich in information and the dual perspective of patients and professionals revealed similar information on the benefits of HM in deprived patients in EDs. While not designed as an implementation study based on classical models, our results answered to several points central to implementation analyses: feasibility, acceptability, appropriateness, penetration, identification of barriers and constraints, and sustainability [51, 52]. However, we did not address the issues of adoption, fidelity and implementation cost. We collected data before the results of the quantitative component of the M2PRUSSE study which will evaluate the efficacy of HM on ED readmissions. If we had waited one more year to obtain the results after the end of the follow-up of the last patients, we would have faced two major methodological drawbacks: recall bias and inability to contact both many patients and ED professionals. The targeted patients are in a vulnerable situation and they often change their postal and telephone addresses. The turnover of ED professionals has been high, especially in the context of the French health emergency crisis, further aggravated by the COVID-19. This context would have made it very difficult, if not impossible, to carry out interviews with ED professionals who had been in contact with the HMrs present at the time of the study.

The success of HMrs’ interventions was achieved by an inclusive approach which did not consider precariousness as a deficit to be compensated, but rather as an obstacle to social participation [53]. The HMrs continued the interventions of health care providers, by encouraging the empowerment of patients through health education. The support offered by the HMrs promoted access to fundamental rights by mobilizing the patient’s individual predispositions, induced an active participation of the patients in a relational alliance. However, this HM intervention requires permanent questioning to ensure that the means of access to autonomy can be mobilized. We must remain cautious, moreover, with the autonomy dynamic, according to which individuals should control all aspects of their lives [54]. The prolonged experience of precariousness can lead persons to favour a status quo strategy and prevent them from participating (e.g., lack of self-esteem, difficulty in projecting themselves) [55]. HM therefore requires building a “power to act” through the potentialities and around the plurality of the persons [56]. Consequently, HM should ensure a network that protects these persons from stigmatization while strengthening their motivation and power to act.

No unavoidable barriers to the implementation of HM in EDs for deprived frequent ED users were identified. However, certain constraints should be taken into account for an efficient implementation of HM. Thus, even if patients and ED professionals expressed their satisfaction and their wish to see HMrs perpetuated, the interviewed HMrs expressed a lack of recognition of their specificity both by patients and ED professionals, which can result in feelings of discomfort, low self-esteem and demotivation. The lack of knowledge of HM by ED professionals may generate an over-investment on the part of ED professionals as well as patients on HMrs, who can appear as the only solution to a precariousness that had not been solved. Second, HMrs can be in distress when faced with psychosocial situations encountered with these deprived patients, in a difficult and stressful environment such as the ED, for which they are generally not initially trained. It is therefore necessary to set up a regular psychological support system. Third, HMrs are in a dual position: on the one hand, they need to be close to patients in order to establish an alliance, on the other hand, they need to be close to ED professionals to collaborate on the patients’ need. In this respect, we can wonder about the objectives of each actor and the meaning that each one gives to the concept of health: a simple absence of illness, a structural, functional, and emotional state compatible with effective life as an individual and as a member of society, … [5]. A framework must therefore be proposed so that HMrs and ED professionals know each other, recognize each other and work towards a common goal of reducing readmissions in deprived persons, but also of equitable health distribution.

Several ED professionals reported that they were faced with intense assignments and high psycho-emotional load on a daily basis, which led to feelings of dissatisfaction, when caring for the deprived frequent ED users. This feeling can contribute to the risk of burn out, which is so prevalent in ED especially since the beginning of the COVID-19 pandemic. A systematic review showed that on average 26% of the ED nurses suffered from burnout, because of work-related factors such as exposure to traumatic events, job characteristics, and organisational variables [57]. Beyond the resolution of the patients’ difficulties, the presence of HMrs was recognized by ED professionals as an improvement and a support in their task. The intervention of HMrs to prevent frequent admissions of deprived patients alongside ED professionals, and the social sharing of emotions with HMrs could prevent some of the burnout of ED professionals, and then enhance their motivation to take charge of deprived frequent ED users. Further implementation study on HM in EDs should explore this dimension which is a key issue of human resources in EDs.

The question of temporality and its very different perception depending on whether it is a patient, an ED professional or a HM, emerged from our analyses as a major point of understanding the issues involved in caring for frequent ED users. According to the ED professionals, deprived patients refer to EDs because they expect prompt care, which is at the heart of their mission as defined by the institution, even if there is not always medical legitimacy. Several statements by ED professionals highlighted the notion of short-term temporality in relation to the emergency in which they work and the importance of the workload as reported by several authors [10, 11]. Similarly, most of patients reported that they referred to EDs to obtain an immediate response to a difficulty with underlying causes. When facing poverty, social exclusion, or socioeconomic insecurity, persons are prone to psychologically leave out the future, and to restrain their time perspective to the present or past, which partly explains why, for many deprived persons, a perceived long delay for medical consultation can be a barrier to seeking care [58]. Their meeting with the ED health professionals is the promise of an immediate response to their demand, even if patients also usually recognize that the roots of their health disorders are deeper. In contrast, HMrs are situated in a longer-term perspective, as they must respond to pressing patient demands, while carrying out repeated interventions with lasting effects and trying to build up a more sustainable relationship with patients. Thus, treatment in EDs is a two-step process: an immediate relationship with time and a second one that requires the creation of a relationship of trust.

Our results contribute to the ongoing debate about the strategies to reduce the readmission rate of frequent ED users, especially those living in precarious conditions [20–23]. Raven et al. put forward that the high inter-study heterogeneity could be explained by the mixed profile of frequent ED users and of strategies tested: case management, care plans, diversion strategies to non-urgent care, printout case notes….[20]. Our findings align with other similar approaches tested in previous research on case management and HCW. Focusing on bio-social and psychological distress, prioritising the inclusivity, health literacy and empowerment dimensions, avoiding being performative and prescriptive are essential parameters when designing interventions targeting frequent ED users living in precarious conditions. They contribute to fill some gaps on how to manage the issue of frequent users of EDs [59]: conditions of adaptation to deprived patients, in particular the taking into account of the psychosocial dimension of deprivation and precariousness, satisfaction of patients and ED professionals as regards implementation and sustainability, profile and training of professionals managing frequent users.

## Conclusion

Health mediation seems a promising solution for saturated EDs to reduce the readmission rate of frequent users living in precarious conditions. At the interface of the patients’ health experience and the medico-social sector, HM could complete the immediate responses to medical needs given in EDs, strengthen the interprofessional networks inside and outside EDs, lighten the psychological burden of ED professionals, alleviate the health-related wandering of deprived patients, improve their overall medical and social care, and impact health literacy to achieve a better empowerment on health and health care in persons living in precariousness. HM could also be integrated in other strategies to reduce the number of repeat admissions to emergency departments to improve health distribution and to fight the increasing social inequalities of health. Future studies should determine the efficiency of HM in reducing the rate of readmission in this vulnerable population and to evaluate their cost-effectiveness.

## Data Availability

The datasets used and/or analysed during the current study are available from the corresponding author on reasonable request.
