# Correction to: Health mediation: an intervention mode for improving emergency department care and support for patients living in precarious conditions

**DOI:** 10.1186/s12913-024-10690-0

**Published:** 2024-02-15

**Authors:** Riwan Naït Salem, Michel Rotily, Themistoklis Apostolidis, Sophie Odena, Aurore Lamouroux, Célia Chischportich, Nicolas Persico, Pascal Auquier

**Affiliations:** 1 Laboratoire de Psychologie Sociale (LPS), Aix Marseille Université, Aix-en-Provence, France; 2 Centre de Santé Universitaire - Espace Santé Aygalades– Assistance Publique Hopitaux de Marseille, Marseille, France; 3 Centre d’Etudes et de Recherche sur les services de santé et la qualité de vie (CEReSS), Aix Marseille Université, Marseille, France; 4 Aix Marseille Univ, CNRS, LEST, Aix-en-Provence, France; 5 SCOP Regards Santé, Marseille, France; 6 Service d’Accueil des Urgences Adultes, Hopital Nord, AssistancePublique Hopitaux de Marseille, Marseille, France

**Correction to:** ***BMC Health Services Research*** **(2023) 23:495**

10.1186/s12913-023-09522-4

In this article the affiliation details for the fourth author (Sophie Odena) were incorrectly given as ‘Laboratoire d’Economie et de Sociologie du Travail (LEST-UMR 7317), Aix Marseille Université, Aix-en-Provence, France’ but should have been ‘Aix Marseille Univ, CNRS, LEST, Aix-en-Provence, France’.

This error is corrected in the author list of this Correction article and the original article has been updated.

